# Reviewed article: Pinheiro TP, Warmling D, Hofelmann DA, Coelho EBS. Completeness, consistency, and duplicity of self-mutilation reports by adolescents in the Notifiable Diseases Information System: evaluation study, Santa Catarina, 2014-2023. Epidemiol Serv Saude. 2026:35;e20250412

**DOI:** 10.1590/S2237-96222026v35e20250412.a

**Published:** 2025-11-28

**Authors:** Denis Moreira

**Affiliations:** 1 Universidade Federal de Alfenas, Alfenas, MG, Brazil Universidade Federal de Alfenas Alfenas MG Brazil

## Peer review A

## Questionnaire

Does the manuscript contain new and significant information to justify publication? Yes

Does the Abstract (Summary) clearly and accurately describe the content of the article? Yes

Is the problem significant and concisely stated? Yes

Are the methods described comprehensively? Yes

Are the interpretations and conclusions justified by the results? Yes

Is adequate reference made to other work in the field? Yes

## Manuscript Structure

Length of article is: Adequate

Number of tables is: Adequate

Number of figures is: Adequate

Please state any conflict(s) of interest that you have in relation to the review of this paper. None

Interest: Excellent

Quality: Excellent

Originality: Excellent

Overall: Excellent

Recommendation: Minor Revision

## Comments

O artigo apresenta uma importante contribuição ao campo da saúde pública, especialmente no que se refere à vigilância de agravos relacionados à saúde mental de adolescentes. A escolha do tema, o delineamento de estudo e a análise crítica dos dados demonstram rigor em uma pesquisa de natureza documental. Sugere-se, para fortalecer o texto: na pág 6, linha 18 fazer uma atualização das questões de gênero com a terminologia vigente LGBTQQICAPF2K+; na linha 33 descrever álcool e outras drogas para não dar a impressão que o álcool não é uma droga e expandir a discussão sobre possíveis estratégias de capacitação profissional e uso de tecnologia para melhorar o registro das notificações.

